# Effect of a farnesyl transferase inhibitor (R115777) on ductal carcinoma *in situ *of the breast in a human xenograft model and on breast and ovarian cancer cell growth *in vitro *and *in vivo*

**DOI:** 10.1186/bcr1395

**Published:** 2006-04-12

**Authors:** Fredrik Wärnberg, Daniel White, Elizabeth Anderson, Fiona Knox, Robert B Clarke, Julie Morris, Nigel J Bundred

**Affiliations:** 1 Breast Biology Group, Christie Hospital NHS Trust, Manchester, UK; 2 Department of Pathology, South Manchester University Hospital, Manchester, UK; 3 Department of Surgery, South Manchester University Hospital, Manchester, UK; 4 Department of Medical Statistics, South Manchester University Hospital, Manchester, UK

## Abstract

**Introduction:**

The ras pathway is essential for cell growth and proliferation. The effects of R115777, a farnesyl transferase inhibitor, were investigated in cancer cell lines expressing varying levels of growth factor receptors and with differing ras status. Effects on tumour xenografts and human ductal carcinoma *in situ *(DCIS) of the breast in a xenograft mouse model were also tested.

**Method:**

*In vitro*, the concentrations required to reduce cell numbers by 50% (50% inhibitory concentration) were established (MDA-MB231, MCF-7, MCF-7/HER2-18, BT-474, SK-BR3 and SKOV3). Human DCIS was implanted in nude mice or, in separate experiments, cultured cells were injected (MDA-MB231, MCF-7/HER2-18, SKOV3) and allowed to form tumours. Proliferation and apoptosis were determined by immunohistochemistry in xenografts and cell tumours.

**Results:**

The 50% inhibitory concentrations varied a hundred-fold, from 39 nmol/l (± 26 nmol/l) for SKBR3 to 5.9 μmol/l(± 0.8 μmol/l) for MDA-MB231. In MCF-7/HER2-18 and SKOV3 cells the levels of tumour growth inhibition were approximately 85% and 40%, respectively. There was a significant decrease in the cell turnover index (CTI; proliferation/apoptosis). In MDA-MB 231 with activated k-ras no inhibition was observed. In treated DCIS xenografts proliferation decreased and apoptosis increased. The CTI ratio between the start and 1 and 2 weeks of treatment were 1.99 and 1.50, respectively, for controls and 0.85 (*P *= 0.005) and 0.75 (*P *= 0.08) for treated xenografts.

**Conclusion:**

Treatment with the farnesyl transferase inhibitor reduced cell growth *in vitro *and cell tumour growth *in vivo*. In DCIS treatment resulted in a reduced CTI. R115777 is a promising treatment for breast cancer but the relation between effect and growth factor receptor and ras status has to be established.

## Introduction

RAS pathway activity is critical for tumour growth [1]. In breast cancers, activity of the ras signalling pathway appears to be increased chiefly via activation of wild-type ras proteins by upstream growth factor receptors such as the epidermal growth factor receptor and c-erbB-2 (HER2) [2,3]. Expression of these receptors is increased in 30–40% of all breast cancers [4]; furthermore, over-expression is indicative of a more aggressive phenotype [5,6].

Point mutations in ras genes result in over-activated ras proteins. The incidence of ras mutations in human tumours overall is about 30%, whereas the incidence of activating ras mutations in breast cancers is low, at only a few percent [7-9]. The ras pathway is activated by a variety of extracellullar factors [10] and aberrant function of the ras signal transduction pathway is thought to be common in breast cancer [2]. Preclinical data have suggested that ras inhibition has an inhibitory effect on growth in cell lines that express both mutated and wild-type ras [11-13].

The ras proteins play an important role in signal transduction and cellular transformation [14]. To function in signal transduction and activation of downstream pathways ras must attach to the plasma membrane where it can interact with membrane receptors and downstream effectors such as RAF and phosphatidylinositol 3-kinase (PI3K). Activation of the RAF-MAPK (mitogen-activated protein kinase) pathway stimulates proliferation in the cell, and activation of the PI3K-PKB (protein kinase B)/Akt pathway is related to increased cell survival [15]. Ras is synthesized as a pro-peptide (pro-ras). One way to target ras activity is to inhibit the enzyme farnesyl transferase, which catalyzes a major step in the modification of pro-ras. Farnesylation makes the ras protein more hydrophobic, and therefore it becomes possible for the protein to attach to the plasma membrane [10]. Several different farnesyl transferase inhibitors (FTIs) have been developed. The drug investigated in this study (R115777) is an imidazole-based FTI that acts as a competitive inhibitor of the carboxyl-terminal tetrapeptide CAAX of ras [16-19].

In the present study we tested the effects of the FTI R115777 on growth of breast and ovarian cancer cell lines expressing varying levels of HER2 and epidermal growth factor receptor and with differing ras status, and on ductal carcinoma *in situ *(DCIS) of the breast in a human xenograft model. Widespread DCIS was previously used for implantation in experiments similar to this study [20,21]. The take rate of DCIS xenografts has been shown to be high; furthermore, DCIS express HER2 in 70% of high-grade DCIS lesions [22,23], which makes DCIS suitable for studies targeting the ras pathway.

In this study we have shown that treatment with the FTI R115777 reduced cell growth *in vitro *and cell tumour growth *in vivo*. In human DCIS xenografts treatment resulted in a reduced cell turnover index (CTI).

## Materials and methods

### Patients

Participants in this study were women who attended the Nightingale Breast Screening Assessment Centre or the Symptomatic Breast Clinic at the University Hospital of South Manchester, UK during the period from April 2002 to April 2003. Women were included in the study if they had mammograms showing widespread microcalcifications indicative of DCIS, with or without an invasive component, and histopathological confirmation of the diagnosis by core biopsy before surgery (*n *= 14). Written consent was obtained, and only those women undergoing mastectomy were included. A consultant breast pathologist subsequently reviewed all tissue samples. Approval to remove tissue from pathological samples in this study was granted by the South Manchester Research Ethics Committee.

### Animals

Athymic female nude mice (BALB/c nu/nu, aged 6–8 weeks) were purchased and allowed to acclimatize for 1–2 weeks before experiments were started. The mice were housed under conventional conditions with a 12-hour cycle of light and dark in filter top cages (*n *= 5–6/cage) and given free access to normal feed, water and bedding during the experiments. All care of the animals and surgical procedures were performed in accordance with Home Office Regulations and the UK Scientific Procedures Act (1986).

### Treatment of ductal carcinoma *in situ *tissue samples and xenografts

A 1–2 cm^3 ^specimen of breast tissue from the area with microcalcifications was taken at the time of surgery and handled as described previously [20,21]. The specimen was dissected into 2 × 2 × 1 mm pieces and a random collection of 5–10 pieces was withdrawn as day 0 samples and processed for histology. The tissue was collected well away from any areas showing signs of invasive cancer on the mammogram, and in our day 0 xenografts we found no signs of invasive cancer. The remaining xenografts were implanted into 5–30 mice, depending on tissue volume. Each mouse received tissue from one patient only and implantation was completed within 90 min of removal of the tissue from the patient. Eight xenografts (in pairs) were implanted subcutaneously in four sites symmetrically in the flanks. After 2 weeks one pair of xenografts was removed and treatment was started (day 14). Another pair of xenografts was removed after 7 days of treatment (day 21), and the remaining xenografts were removed after 14 days of treatment (day 28) at the end of the experiment. After retrieval, the xenografts were immediately fixed in buffered formalin for 24 hours at 4°C and then stored in 70% ethanol before paraffin embedding.

### Treatment

Treatment with R115777 (50 mg/kg or 100 mg/kg; Johnson and Johnson Pharmaceutical Research and Development, Raritan, NJ, USA) or control vehicle (20% β-cyclodextrin) was given as a gavage twice daily from Monday to Friday and once daily during weekends. Treatment was given over 14 days in the experiments with human DCIS. In the experiments with implanted cell tumours the duration of treatment varied between 8 and 29 days in different experiments, depending on how fast the tumours grew. We chose the doses 50 mg/kg and 100 mg/kg, based on data from previous experiments with R115777 [12].

### Ras activity *in vitro *and *in vivo*

#### Cell culture

The human breast cancer cell lines MDA-MB231, MCF-7, MCF-7/HER2-18, BT-474 and SK-BR3 and the human ovarian cancer cell line SKOV3 were obtained from the American Type Culture Collection (Rockville, MA, USA) and grown and subcultured according to their instructions. The breast cancer cell lines were chosen based on their different expression of growth factor receptors, and the SKOV3 ovarian cell line was used as a model for oestrogen receptor (ER) negative and wild-type ras cancer. Data concerning growth factor expression, ER expression and ras protein status were collected from the literature and are given in Table 1.

#### Assessment of cell growth *in vitro*

The effect of R115777 on cell growth was used to establish the concentrations required to reduce cell numbers by 50% (50% inhibitory concentration [IC_50_]). Cells were plated in 96-well plates in growth medium containing 2–5% foetal calf serum (FCS), and after 24 hours the medium was discarded and replaced with medium containing varying concentrations of R115777 (0, 1 nmol/l, 10 nmol/l, 100 nmol/l, 500 nmol/l, 1 μmol/l, 5 μmol/l and 10 μmol/l). The plated number of cells and the concentration of FCS were established for each cell line in separate experiments to find suboptimal growing conditions for use in the following experiments with R115777. Treatment was continued until untreated cells reached confluence. Estimation of cell number was done by means of the sulphorhodamine B colorimetric assay. Three separate experiments were conducted for each cell line, in which each concentration of R115777 was tested in quadruplicate.

#### Assessment of tumour growth in nude mice

Cells (2–4 × 10^6^) in a 200 μl solution of growth medium containing 10% FCS were injected subcutaneously into each flank of each mouse and allowed to form tumours. In the experiments involving the oestrogen-dependent MCF-7/HER2-18 cell line, oestrogen pellets (2 mg) were implanted subcutaneously at the base of the tail of the mice at the time of cell injection [24]. Treatment with R115777 50 mg/kg or 100 mg/kg or control vehicle (20% β-cyclodextrin) was begun when measurable tumours were formed in about 75% of injection sites. Only tumours that were measurable at the start of treatment were included in the analyses. Tumours were measured with a calliper twice weekly and tumour volume was calculated using the following formula: tumour volume = (width)^2 ^× (length/2). Each experiment was terminated and all tumours were removed when any of the mice developed a tumour burden of more than 1 cm^3^. Tumours were divided and processed for immunohistochemistry or were stored in liquid nitrogen. The growth inhibitory effect of R115777 was calculated using the following formula: 1 - ([D - C]/[B_median _- A_median_]) × 100%, where D is the final tumour volume after R115777 treatment, C is the tumour volume before R115777 treatment, B is the median final tumour volume among control tumours after treatment, and A is the median tumour volume among control tumours before treatment. For each of the cell lines two separate experiments were conducted and the median growth inhibition among all R115777-treated tumours was calculated.

### Assessment of proliferation index, apoptotic index and cell turnover index

Sections of DCIS xenografts and cell tumours were scored for proliferation after Ki67 immunohistochemical staining (MIB-1; Coulter-Immunotech, Luton, UK). At least 300 cells in the xenografts and 1,000 cells in the cell tumours were counted at a magnification of ×40. To assess apoptosis in the xenografts, we sought morphological evidence of apoptosis using haematoxylin and eosin stained sections. In the cell tumours immunohistochemistry (TdT-mediated dUTP-fluorescence nick end labelling) was used. At least 1,000 cells were counted at a magnification of ×40. Proliferation and apoptotic indices were both expressed as a percentage of apoptotic or stained cells. The immunohistochemistry methodology and apoptotic scoring was described previously [21,25-27]. A CTI (proliferation index/apoptotic index) [28] was calculated for each individual xenograft and cell tumour. All counting was performed without knowledge of treatment group.

### Assessment of farnesyl transferase inhibition *in vitro *and *in vivo*

Western blots were performed on MCF-7 and MCF-7/HER2-18 cells treated with different doses of R115777 and on MCF-7/HER2-18 tumours from the *in vivo *experiments. Protein extracts were prepared from cell pellets or tumours after thawing the cryopreserved samples and washing them with phosphate-buffered saline. The cells were lysed in ice-cold radioimmunoprecipitation assay buffer (50 mmol/l Tris-HCl [pH 7.5], 150 mmol/l NaCl, 0.1% Triton X-100, 0.5% deoxycholate, 0.1 mmol/l ethyleneglycoltetracetic acid, and 0.1 mmol/l EDTA) with protease inhibitors for 5 minutes on ice. The protein concentration of each lysate was quantified using a colorimetric assay (BCA Protein Assay; Pierce, Rockford, IL, USA) and 50 μg total protein was loaded on an 8% SDS polyacrylamide gel. The Western blots were transferred to polyvinyledene difluoride membrane and probed with a mouse monoclonal antibody against HDJ2 (KH2A5.6; NeoMarkers, Fremont, CA, USA) [29]. Bound antibody was detected using a horseradish peroxidase conjugated secondary antibody and visualized by treating the membrane with enhanced chemiluminescence reagents (Amersham Pharmacia Biotech Inc., Piscataway, NJ, USA) and exposing it to film. Detection of an unfarnesylated and a farnesylated band of the HDJ2 protein represents conclusive evidence of farnesyl transferase inhibition.

### Statistical analysis

The IC_50_s are presented as the mean value of three separate experiments with the corresponding standard errors of the mean. Tumour growth inhibition is given as a percentage. The percentage with interquartile range is based on the median growth inhibition among treated tumours relative to the median control tumour growth. Statistical significance of tumour growth inhibition was tested by comparing individual tumour growth in cubic millimetres between treated and control tumours using the Mann-Whitney *U* test.

Comparisons between the R115777 treated and control samples in the DCIS xenograft experiments were made 'within-patient' using a repeated analysis of variance model with patient and group (R155777 versus control) as factors. This statistical model matched the R11577 and control readings from mice transplanted from the same women. Data were restricted to those mice with tissue from women with readings available from both R115777 and control mice. Throughout the analysis, 'mouse' was taken as the unit of analysis and only data from mice with data from both day 14 and day 21 or day 28 were included, because we looked at change over time in terms of proliferation, apoptosis and CTIs. Data are presented as the geometric means with their 95% confidence intervals. The significance level was set at 5% and all tests were two sided. Statistical analysis was performed using STATA software (STATA Corporation, College Station, TX, USA).

## Results

### Cell growth

IC_50 _for R115777 varied a hundred-fold, from 39 nmol/l and 46 nmol/l for SK-BR3 and MCF-7 to 2.7 μmol/l and 5.9 μmol/l for SKOV3 and MDA-MB231 cell lines, respectively, when grown *in vitro *(Table 1). The cell line with the mutated k-ras, namely MDA-MB231, had the highest IC_50_, whereas in the cell lines with wild-type ras the drug was effective at lower doses.

**Figure 1:**
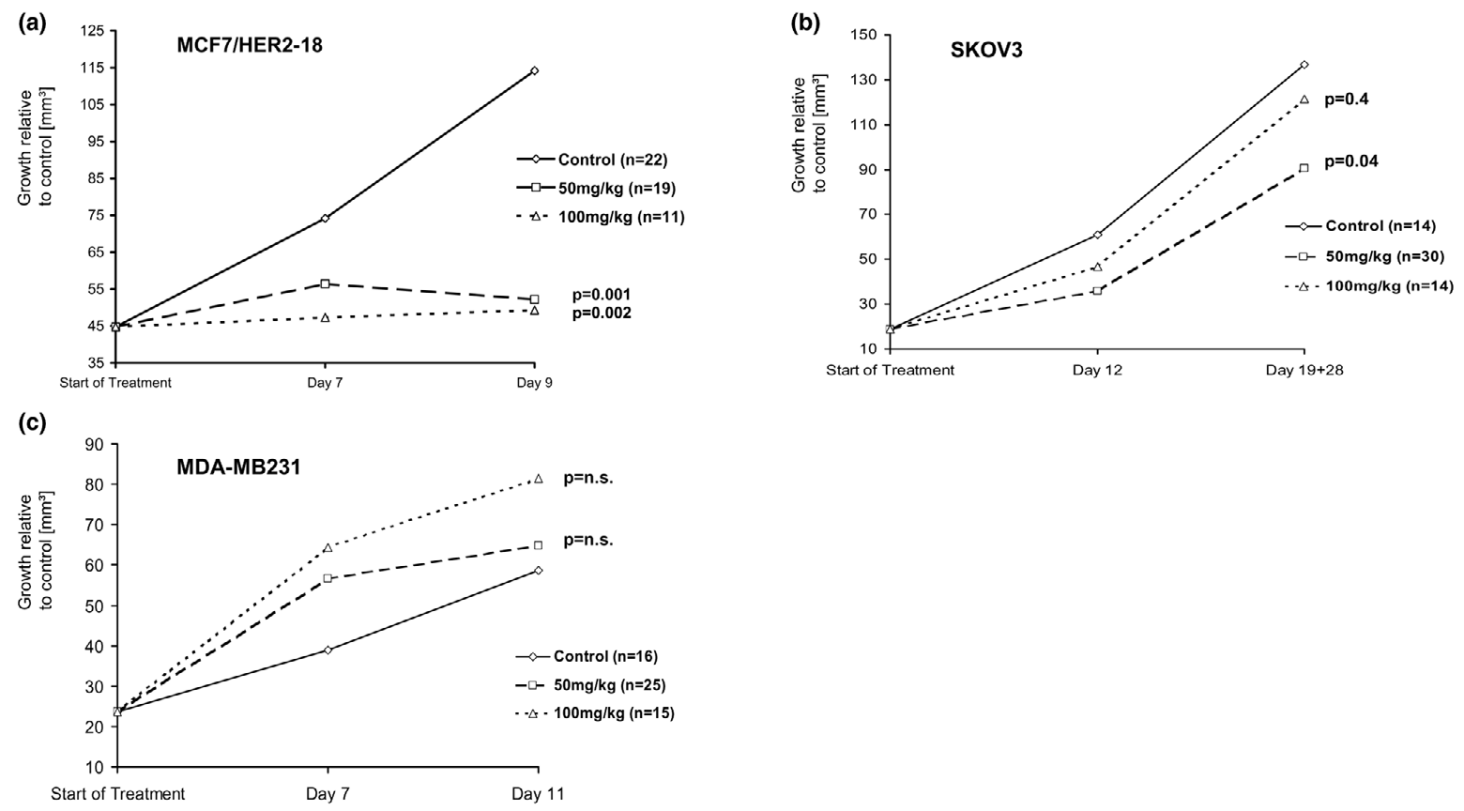
Tumour growth inhibition. **(a) **Tumour growth inhibition in MCF-7/HER2-18 tumours grown in athymic mice treated with R115777 50 mg/kg or 100 mg/kg. Median tumour volume in treated relative to control animals is given in cubic millimetres. **(b) **Tumour growth inhibition by R115777 in SKOV3 tumours. **(c) **Tumour growth inhibition by R115777 in MDA-MB231 tumours.

In mice, growth of MCF-7/HER2-18 tumours was inhibited by R115777 50 mg/kg (*n *= 19) and 100 mg/kg (*n *= 11) by 80.8% (interquartile range 56.4–99.0%; *P *= 0.001) and 95.9% (68.2–110.1%; *P *= 0.02), respectively, compared with control tumours (*n *= 22; Figure 1a and Table 1). The proliferation index was lower in the treated tumours; for R115777 50 mg/kg it was 69.6% (63.4–74.8%; *P *= 0.003) and for R115777 100 mg/kg it was 65.5% (62.0–70.1%; *P *< 0.0001) as compared with 77.7% for the control tumours (74.4–81.1%; Table 2). The apoptotic index was higher in the treated tumours; for R115777 50 mg/kg it was 1.5% (1.2–1.6%; *P *= 0.04) and for R115777 100 mg/kg it was 1.6% (1.4–1.9%; *P *= 0.003) as compared with 1.2% in the control tumours (0.9–1.5%). The CTIs for tumours treated with R115777 50 mg/kg and 100 mg/kg were 48.7 (41.6–57.4; *P *= 0.0009) and 38.0 (30.1–43.3; *P *< 0.0001), respectively, and these values were statistically significantly reduced as compared with the CTI of 61.6 in controls (55.5–79.5; Table 2).

Growth of SKOV3 tumours was inhibited by R115777 50 mg/kg (*n *= 30) and 100 mg/kg (*n *= 14) by 60.1% (32.3–83.9%; *P *= 0.04) and 20.4% (-65.7 to +38.3%; *P *= 0.4), respectively, as compared with that in control tumours (*n *= 14; Figure 1b and Table 1). The proliferation index was lower in treated tumours; for R115777 50 mg/kg it was 34.1% (26.5–44.4%; *P *= 0.009) and for R115777 100 mg/kg it was 40.1% (33.1–44.0%; *P *= 0.08) as compared with 46.6% in the control tumours (37.6–55.0%). The apoptotic index did not differ between treated tumours; for R115777 50 mg/kg it was 0.49% (0.4–0.7%; *P *= 0.11), for R115777 100 mg/kg it was 0.48% (0.4–0.8%; *P *= 0.18) and it was 0.35% in the control tumours (0.3–0.5%). The CTIs for tumours treated with R115777 50 mg/kg and 100 mg/kg were 67.5 (45.6–96.1; *P *= 0.004) and 81.0 (38.6–110.2; *P *= 0.05), respectively; these values were statistically significantly reduced as compared with the CTI of 125.4 in controls (87.1–188.9; Table 2).

Growth of k-ras mutated MDA-MB231 tumours was not inhibited by R115777 50 mg/kg (*n *= 25) and 100 mg/kg (*n *= 15). Instead, the growth in the treated tumours was increased by 68.8% (13.8–284.1%; *P *= 0.08) and 91.2% (2.8–328.8%; *P *= 0.09), respectively, relative to control tumours (*n *= 16; Figure 1c and Table 1). The proliferation index was higher, although not statistically significantly higher, in the treated tumours; for R115777 50 mg/kg it was 68.2% (63.3–71.5%; *P *= 0.24) and for R115777 100 mg/kg it was 72.4% (66.4–78.4%; *P *= 0.06) as compared with 57.9% in the control tumours (54.1–71.4%). The apoptotic index was similar in treated and control tumours; for R115777 50 mg/kg it was 0.4% (0.3–0.6%; *P *= 0.98), for R115777 100 mg/kg it was 0.4% (0.3–0.7%; *P *= 0.75) and for controls it was 0.4% (0.3–0.6%). The CTIs for tumours treated with R115777 50 mg/kg and 100 mg/kg were 180.1 (115.9–205.4; *P *= 0.32) and 180.8 (90.5–227.7; *P *= 0.37), respectively, and the CTI for controls was 106.0 (85.8–191.0; Table 2).

In the xenograft experiments no difference in body weight was seen between R115777 and controls; neither was there any difference in the number of deaths seen between treated and control animals.

### Ductal carcinoma *in situ*

Tissue from 14 women with widespread DCIS was implanted. In four of the cases no DCIS was found in the day 0 xenografts or in the implanted xenografts, and these women were excluded from further analyses. The median age among the remaining 10 women with implanted DCIS was 53 years (range 39–72 years). Nine out of the 10 women had foci of invasive cancer in their mastectomy specimen according to the final histopathological report. Nine lesions were nuclear grade III and the remaining lesions were nuclear grade II. The grading was done on the *in situ *part only in lesions with invasive cancer. Accordingly, the data presented on proliferation, apoptosis and CTI are from the 10 experiments with implanted DCIS. Four experiments were conducted with the dose R115777 50 mg/kg and six with 100 mg/kg. Eight out of the 10 cases of DCIS were ER positive (for instance, >5% stained cells). Seven of the cases were clinically regarded as positive for HER2 (2+ or 3+) and three were regarded as negative (0 or 1+).

Fifty-four out of 80 (67.5%) day 0 xenografts contained ducts with DCIS. The harvested xenograft pairs contained DCIS in 44.8% (493/1100). Accordingly, the overall take rate was calculated at 66.4%.

The proliferation and apoptotic indices and CTI from the DCIS experiments are presented in Table 3. There was decreased proliferation in the xenografts treated with R115777. The ratios of proliferation indices between day 14 and day 21 were 1.38 for controls (*n *= 65) and 0.75 for treated (*n *= 61) xenografts, respectively (*P *= 0.04). The ratios between day 14 and day 28 were 1.22 for controls (*n *= 75) and 0.78 for treated (*n *= 66) xenografts, respectively (*P *= 0.06).

Apoptosis increased in the treated DCIS xenografts. The ratios between day 14 and day 28 were 0.83 for controls (*n *= 95) and 1.06 for treated (*n *= 104) xenografts, respectively (*P *= 0.005).

Overall, CTI decreased in treated xenografts. The ratios of CTIs between day 14 and day 21 were 1.99 for controls (*n *= 44) and 0.85 for treated (*n *= 49) xenografts, respectively (*P *= 0.005). The ratios between day 14 and day 28 were 1.50 for controls (*n *= 67) and 0.75 for treated (*n *= 62) xenografts, respectively (*P *= 0.08). The results of proliferation and apoptosis in the experiments with the different doses of R115777 pointed in the same direction, except for an unchanged apoptotic index after 7 days of treatment in the lower dose group (data not shown).

There was no statistically significant difference in body weight loss/gain or number of deaths between treated and control animals in the DCIS xenograft experiments.

### Farnesyl transferase inhibition

**Figure 2:**
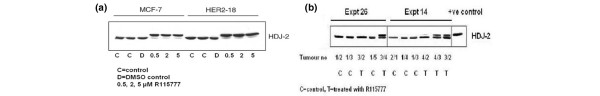
Farnesyl transferase inhibition *in vitro *and *in vivo*. **(a) **Detection of an unfarnesylated and a farnesylated band of the HDJ2 protein by Western blot technique on cell pellets treated with different doses of R115777 and controls. All treated cell pellets exhibit a high proportion of unfarnesylated protein. **(b) **Detection of an unfarnesylated and a farnesylated band of the HDJ2 protein by Western blot technique on MCF-7/HER2-18 cell tumours treated with R115777 or control vehicle. Tumours were selected from those tumours treated with R115777 exhibiting a good response and control tumours with no response on tumour growth. In four out of five treated tumours an unfarnesylated protein was detected.

In all MCF-7 and MCF-7/HER2-18 cell samples treated with different doses of R115777 *in vitro*, an unfarnesylated ras band was detected with Western blot technique. This unfarnesylated ras band was not detected in any of the untreated cell samples (Figure 2a). The cell tumours chosen in this analysis were MCF-7/HER2-18 tumours showing good response on R115777 treatment and MCF-7/HER2-18 control tumours with uninhibited growth. This selection of tumours was made in an attempt to establish proof of farnesyl tranferase inhibition *in vivo*. In four out of five treated MCF-7/HER2-18 tumours an unfarnesylated ras band was detected, whereas in six out of six control tumours no unfarnesylated ras was detected (Figure 2b).

## Discussion

In this study we demonstrated reduced tumour growth *in vivo *and reduced cell growth *in vitro *in human cancer cell lines with wild-type ras and varying levels of growth factor receptors, when treated with the FTI R115777. We have shown that the effect on growth is related to a reduced CTI in the cancer cell lines tumours *in vivo *and that the CTI was statistically significantly reduced in human DCIS treated by the FTI after 1 week. The effect on the CTI was due to both an antiproliferative and a pro-apoptotic effect.

To study the effect of an FTI, we conducted a series of experiments: first, the *in vitro *studies on different human cancer cell lines; second, *in vivo *studies of tumour growth inhibition in selected cell lines; and, finally, studies of human DCIS implanted directly from the operating theatre into nude mice in a model earlier used in our department. The ras pathway is activated by a variety of extracellullar factors, and aberrant function of ras signal transduction is not only due to ras mutations. We could not assess activation of the ras signalling pathway, but chose to study the downstream effects of the FTI, measured as change in apoptosis and proliferation. We also tried to relate the effect of the FTI to growth factor receptor status and ras status. Furthermore, by showing an unfarnesylated protein in treated cell pellets and cell line tumours, we demonstrated the effect on farnesylation by the FTI R115777.

Strikingly, we found an effect on proliferation and apoptosis that was consistent in the *in vivo *studies of cell tumours expressing wild-type ras (MCF-7/HER2-18 and SKOV3) and in human DCIS. The results of growth inhibition *in vitro *were in accordance with the *in vivo *results (for instance, the largest effect on tumour growth *in vivo *was seen in the cell line with the lowest IC_50 _*in vitro*). No antiproliferative effect and no pro-apoptotic effect were seen in the k-ras mutated MDA-MB231 tumours treated with the FTI, and no tumour growth inhibition resulting from treatment was observed in this cell line. The MDA-MB231 cell line also had the highest IC_50_. Other FTIs have been shown to inhibit both mutated and wild-type ras [30,31], but a relative resistance to FTIs in k-ras mutated cell lines has also been reported [32,33]. Although we were unable to establish the ras status of the DCIS lesions in our human DCIS xenograft experiments, the majority (70%) were HER2 positive and would be expected to have activated wild-type ras. We found no areas of invasive breast cancer in our day 0 xenografts. However, if some mixture of invasive cancer cells had occurred in the xenografts, then this should have been evenly distributed between controls and treated animals, therefore causing no bias.

The cell lines considered in our *in vitro *experiments were selected to represent different expressions of growth factor receptor and ERs. We were unable to detect a clear relationship between any of the factors and the effect of the FTI. For example, the IC_50_s in the MCF-7 and in the MCF-7/HER2-18 were not significantly different in the *in vitro *experiments. The MCF-7/HER2-18 is a MCF-7 cell line with 45-fold over-expression of the HER2 receptor caused by transfection with full-length HER2 cDNA [34]. However, in an experiment similar to ours, in which cell line tumour growth inhibition was studied and the same FTI was used, Kelland and coworkers [12] identified inhibition of about 60–70% in treated MCF-7 cell line tumours. This was less than the inhibition of approximately 80% we found on the MCF-7/HER2-18 tumours, using the same dose of R115777. This might be an indication that HER2 expression plays a role in the response to farnesyl transferase *in vivo*. The effect on the HER2 transfected MCF-7 cell line tumours was also considerably more pronounced than the effect on the SKOV3 cell tumours, and this was also reflected in a 10-fold lower IC_50_. Other studies [11,13] also were unable to show a clear relation between markers such as growth factor receptors and ras status, and the effect of FTIs. As in the work by Kelland and colleagues, we found no dose respond effect with the chosen doses, namely 50 and 100 mg/kg. The mechanism might be a threshold effect or due to saturable drug pharmacokinetics [12].

We did not study the effect of the FTI in subgroups of DCIS (for example, ER positive/negative or HER2 positive/negative). We stratified by treatment dose of R115777, but it was not possible to compare the results between the two different doses because the number of xenografts was too small after stratification. However, all results of proliferation and apoptotic change in the different groups pointed in the same direction, except for an unchanged apoptotic index after seven days of treatment in the lower dose group.

Clinical studies of R115777 in phase I trials have shown that a reversible myelosuppression is the dose-limiting toxicity of the drug [35,36]. In a phase II study of continuous and intermittent dosing of R15777 in women with advanced breast cancer [37], partial response or stable disease was demonstrated in about 20% of treated women. Forty-one of the primary tumours in the study conducted by Johnston and coworkers [37] were analyzed for ras mutations and only one tumour had an h-ras mutation. No correlation between response and ER and/or HER2 expression was seen in the study. No clinical studies involving FTIs in patients with DCIS have been performed, and knowledge on ras status in DCIS lesions is sparse. In one study [38] two out of 25 DCIS lesions over-expressed wild-type k-ras, and in another study [39] four out of five DCIS patients expressed detectable plasma levels of wild-type ras, but no ras mutations were found in these studies.

We have shown that the FTI was effective in some tumours and not in others. In the future, it might be possible to identify those tumours that will benefit from treatment with a FTI. A series of new signal transduction inhibitors (STIs) are under development [40]. By gaining a better understanding of how different interactions in normal or upregulated or downregulated signal pathways work, we hopefully will be able to tailor breast cancer treatment to individual patients. Furthermore, data suggest that combining STIs and endocrine therapy may be more effective than an STI alone [41-43]. There is a lack of correlation between preclinical results with FTIs and results from clinical studies. Inhibition of farnesylation has not been clearly correlated to tumour inhibition in cancer patients [44]. There is a need to identify the targets of the FTIs in the preclinical setting if we are to understand the clinical results and be able to select patients in future studies. Breast cancer is potentially a suitable tumour type for study because it is possible to perform experiments similar to ours in which human tumours can be tested in a xenograft model.

The exact mechanism of action FTIs on cancer cells remains unknown. Other ras-independent mechanisms of action might play a role in the antitumour effect, including inhibition of enzymes other than the farnesyl transferases [44,45]. We have clearly demonstrated inhibition of farnesylation *in vitro *in the present study. We also demonstrated an effect on farnesylation in cell tumours, with good response to R115777 treatment. However, the effect was not as clear as in the *in vitro *setting and varied among the treated tumours. This indicates that mechanisms other than farnesyl transferase inhibition are involved in tumour growth inhibition.

## Conclusion

Treatment with the FTI reduced cell growth *in vitro*. Also, cell tumour growth *in vivo *was reduced, and we showed increased apoptosis and decreased proliferation, resulting in reduced CTI. In human DCIS xenografts treatment also resulted in a reduced CTI. The FTI R115777 is a promising new treatment for breast cancer. The type of tumours that respond to farnesyl transferase inhibition must be established before the treatment can become clinically useful. We are planning further investigations into the mechanisms of action of the FTIs and how they affect the different pathways that regulate cell survival and proliferation.

## Abbreviations

CTI = cell turnover index; DCIS = ductal carcinoma *in situ*; ER = oestrogen receptor; FCS = foetal calf serum; FTI = farnesyl transferase inhibitor; IC_50 _= 50% inhibitory concentration; PI3K = phosphatidylinositol 3-kinase; STI = signal transduction inhibitor.

## Competing interests

The research was partly sponsored by Johnson and Johnson Pharmaceutical Research and Development, New Jersey, USA.

## Authors' contributions

FW participated in the design of the study, carried out the cell and tumour experiments, obtained patient consent and collected human DCIS tissue, performed part of the statistical analysis and drafted the manuscript. DW participated in the design of the study, carried out the cell and tumour experiments, and carried out the immunoassays and Western blots. EA participated in the design and coordination of the study. FK participated in the design of the study, classified the human DCIS tissue and helped with the counting of proliferation and apoptosis in tumour cells. RBC participated in the design and coordination of the study. JM participated in the design of parts of the study and performed the statistical analysis. NJB participated in the design and coordination of the study and helped to draft the manuscript. All authors read and approved the final manuscript.

## Figures and Tables

**Table 1:** Results from cell and cell tumour experiments

Cell line	IC_50 _(mean ± SEM)	Tumour growth inhibition *in vivo *by R115777		Ras statusa	ER^a^	EGFR^a^	HER2^a^
						
		50 mg/kg	100 mg/kg				
SK-BR3	39.0 ± 25.8 nmol/l	-	-	Wild-type	-	+	++
MCF-7	46.0 ± 5.0 nmol/l	-	-	Wild-type, over-expression of n-ras	++	+	(+)
MCF-7/HER2-18	60.3 ± 117 nmol/l	80.8%	95.9%	Wild-type, over-expression of n-ras	++	+	+++
BT474	195 ± 119 nmol/l	-	-	Wild-type	+	-	++
SKOV3	2.7 ± 0.4 μmol/l	60.1%	20.4%	Wild-type	-	++	+++
MDA-MB231	5.9 ± 0.8 μmol/l	-68.8%	-91.2%	Mutated k-ras	-	+++	+

The table shows the concentration of R115777 required to reduce cell numbers by 50% (50% inhibitory concentration [IC_50_]), inhibition by R115777 at two different doses in tumours grown in athymic mice, and receptor and ras status for the different cancer cell lines. ^a^Data collected from the literature [4,12,46]. EGFR, epidermal growth factor receptor; ER, oestrogen receptor; SEM, standard error of the mean.

**Table 2:** Apoptosis and proliferation in cell tumour experiments

Cell line	Control	Treated R115777	
		
		50 mg/kg	100 mg/kg
MCF-7/HER2-18			
Ki67 (%)	77.7 (74.4–81.1)	69.6** (63.4–74.8)	65.5**** (62.0–70.1)
TUNEL (%)	1.2 (0.9–1.5)	1.5* (1.2–1.6)	1.6** (1.4–1.9)
CTI	61.6 (55.5–79.5).	48.7*** (41.6–57.4)	38.0**** (30.1–43.3)
SKOV3			
Ki67 (%)	46.6 (32.0–63.8)	34.1** (20.3–57.2)	40.1 (26.6–48.8)
TUNEL (%)	0.35 (0.1–0.8)	0.49 (0.1–1.0)	0.48 (0.1–1.4)
CTI	125.4 (87.1–188.9).	67.5**(45.6–96.1)	81.0* (38.6–110.2)
MDA-MB231			
Ki67 (%)	61.7 (54.1–74.6)	69.3 (56.1–78.0)	72.4* (43.8–83.1)
TUNEL (%)	0.55 (0.3–0.9)	0.45 (0.3–0.6)	0.31* (0.1–0.4)
CTI	106.0 (85.8–191.0).	180.1 (115.9–205.4)	180.8 (90.5–227.7)

Effect of R115777 on proliferation (Ki67), apoptosis (TdT-mediated dUTP-fluorescence nick end labelling [TUNEL]) and cell turnover index (CTI) in cell line tumours grown in athymic nude mice. Values are expressed as median values (interquartile range). *P < 0.05, **P < 0.01, ***P < 0.001, ****P < 0.0001.

**Table 3:** Results from experiments conducted in human DCIS xenografts

Index	Day 0	Day 14		Day 21		Day 28	
		
		Control	R115777	Control	R115777	Control	R115777
Overall effect							
Ki67 (%)	27.1 (19.3–32.9)	9.4 (7.2–12.2)	10.3 (7.7–13.9)	13.2 (9.2–18.9)	7.5* (5.4–10.6)	11.8 (7.7–18.2)	8.4 (7.2–8.2)
Apoptosis (%)	3.7 (3.5–4.2)	2.8 (1.5–5.2)	3.3 (2.1–5.1)	2.7 (1.8–4.1)	3.2 (2.0–5.4)	2.5 (1.5–3.9)	3.8** (2.7–5.2)
CTI	6.2 (5.2–9.1)	2.8 (1.5–5.1)	3.5 (2.0–6.3)	6.2 (3.8–10.3)	2.6** (1.4–5.0)	3.9 (2.6–6.1)	2.4 (1.8–3.1)
R115777 100 mg/kg							
Ki67 (%)	27.1 (19.3–32.9)	8.4 (5.8–12.3)	8.7 (6.1–12.3)	12.8 (7.6–21.5)	6.2*** (4.5–8.7)	12.1 (6.5–22.4)	8.7 (7.2–10.5)
Apoptosis (%)	3.7 (3.5–4.2)	3.3 (1.2–9.1)	3.7 (1.8–7.5)	3.0 (1.8–5.2)	3.9 (2.1–7.4)	2.9 (1.4–6.0)	4.1 (2.5–6.7)
CTI	6.2 (5.2–9.1)	1.8 (1.2–2.7)	2.5 (1.8–3.6)	4.8 (3.8–6.0)	1.9*** (1.4–2.5)	3.2 (2.5–4.1)	2.3 (1.5–3.6)
R115777 50 mg/kg							
Ki67 (%)	27.1 (19.3–32.9)	11.8 (7.0–19.7)	13.9 (10.1–18.9)	14.0 (4.3–45.6)	11.1 (7.2–17.1)	11.1 (3.3–37.3)	7.9 (5.2–12.1)
Apoptosis (%)	3.7 (3.5–4.2)	2.1 (0.8–5.9)	2.6 (1.1–6.1)	2.3 (0.8–6.7)	2.3 (0.6–8.8)	1.9 (0.8–4.3)	3.2* (1.5–6.8)
CTI	6.2 (5.2–9.1)	5.9 (3.1–11.2)	6.1 (2.2–16.7)	9.2 (3.7–23.1)	6.2 (2.1–18.5)	6.3 (2.2–18.6)	2.4 (1.4–4.1)

The overall effect of the farnesyl transferase inhibitor R115777 on proliferation and apoptosis of ducatal carcinoma *in situ *(DCIS) of the breast. Xenografts were implanted into athymic mice on day 0 and left untreated for 14 days. Xenografts were removed on day 14, day 21, and day 28. The mice were gavaged twice daily with either R115777 or control vehicle starting on day 14 after removal of the first pair of xenografts. Proliferation was measured by immunohistochemistry (Ki67) and apoptosis by morphology on haematoxylin and eosin stained slides. Data shown represent the overall effect of R115777 and for both doses separately. Data only include mice for which data were available from day 14 and day 21 or day 28. Data are expressed as geometric means with 95% confidence intervals. Statistical significance was tested for the change between day 14 and day 21 and between day 14 and day 28. *P < 0.05, **P < 0.01, ***P = 0.001. CTI, cell turnover index.
